# Supplementary material for: Estimating the effectiveness of national health insurance in covering catastrophic health expenditure: Evidence from South Korea
Source: PLoS One. 2021 Aug 19;16(8):e0255677. doi: 10.1371/journal.pone.0255677 (PMC8376031; doi:10.1371/journal.pone.0255677)
Supplement: Attachment — Submitted filename: review_4.25.2021.docx [file pone.0255677.s001.docx]

Thank you for the opportunity to review this manuscript. This study uses a large data set the Korea Health Panel Survey (2011-2016) to estimate the effectiveness of insurance in covering CHE. This is an interesting submission that, if you choose to go further, I would recommend some major work. There are several major issues that need to be addressed.

Introduction

1. The authors should conclude this section with the objective or research questions you will address in this paper. A priori hypotheses are not mentioned at all.
2. It is unclear whether the authors want to compare the traditional CHE calculation method with a new approach or try to report the amount of CHE covered by health insurance using two different approaches.
3. The study fails to address how the findings relate to previous research in this area. The authors should rewrite their Introduction and Discussion to reference the related literature.
4. It is not a commentary/opinion article, but an original article. Some sentences need citations.

Methods

5. In the Study Sample section, the study population should be described, including the overall population composition, age range, and size. Reasons for exclusion should include the numbers and percentages excluded.
6. What was the proportion of missing data? How were missing data handled in the analyses?
7. Why not include the 2017 KHPS data as well? Please justify.
8. It is unclear why household income is used for rankings in the concentration index.
9. Which computer package was used?
10. Which ethics committee(s) approved your project?

Results

11. In the first paragraph, please describe the characteristics of the sample, beginning with details of the response rates. The demography of the sample should be described and usually, this will include a table of basic indices, including age and gender. Please include the simple statistics (characteristics of the sample or univariate statistics) before moving to more complex analyses.

Discussion

12. Overall, the discussion needs some more work. Several more points could be elaborated on, including, what are some of the barriers that contribute to the findings?
13. It would be more helpful to supply how findings contrast to others in the literature, implications of your results, and potential mechanisms that contribute to them. How were your findings novel? How were they similar?
14. A possible issue with attributing disparities to household income is that income may not represent economic status. They may have monies that are not taxed and current income may not reflect accumulated assets. This at least needs a discussion.
15. Make sure each of your objectives and research questions is discussed.
