# Supplementary material for: Estimating the effectiveness of national health insurance in covering catastrophic health expenditure: Evidence from South Korea
Source: PLoS One. 2021 Aug 19;16(8):e0255677. doi: 10.1371/journal.pone.0255677 (PMC8376031; doi:10.1371/journal.pone.0255677)
Supplement: Attachment — Submitted filename: Response to Reviewer #2.docx [file pone.0255677.s002.docx]

| Comments of reviewer #2 | Answer |
| --- | --- |
| Overall, this manuscript is very valuable study for Korea and for other countries to indicate the advantages and important roles to reduce health expenses through national health insurance system. I think the authors support the results through using appropriate statistical analytical methods. However, I found that the authors should change some Korean language to English in discussion section. For example, in the sixth paragraph in the discussion section, the authors used Korean language in the two references. Therefore, I recommend that the study should change them to English. I would like to recommend to accept this study with minor pending revision. | Thank you very much for the good evaluation. The references in the Korean language are our major mistake. We have all corrected these. Thank you for reviewing again. |
